# Neutral Dissociation of Pyridine Evoked by Irradiation of Ionized Atomic and Molecular Hydrogen Beams

**DOI:** 10.3390/ijms23010205

**Published:** 2021-12-24

**Authors:** Tomasz J. Wasowicz

**Affiliations:** Division of Complex Systems Spectroscopy, Institute of Physics and Applied Computer Science, Faculty of Applied Physics and Mathematics, Gdansk University of Technology, ul. G. Narutowicza 11/12, 80-233 Gdansk, Poland; tomasz.wasowicz1@pg.edu.pl

**Keywords:** ion–molecule reactions, pyridine, collisions, collision-induced dissociation, luminescence, spectral line shapes, protons, dihydrogen cations, charge transfer, dissociative excitation, dissociative ionization, complex formation

## Abstract

The interactions of ions with molecules and the determination of their dissociation patterns are challenging endeavors of fundamental importance for theoretical and experimental science. In particular, the investigations on bond-breaking and new bond-forming processes triggered by the ionic impact may shed light on the stellar wind interaction with interstellar media, ionic beam irradiations of the living cells, ion-track nanotechnology, radiation hardness analysis of materials, and focused ion beam etching, deposition, and lithography. Due to its vital role in the natural environment, the pyridine molecule has become the subject of both basic and applied research in recent years. Therefore, dissociation of the gas phase pyridine (C_5_H_5_N) into neutral excited atomic and molecular fragments following protons (H^+^) and dihydrogen cations (H_2_^+^) impact has been investigated experimentally in the 5–1000 eV energy range. The collision-induced emission spectroscopy has been exploited to detect luminescence in the wavelength range from 190 to 520 nm at the different kinetic energies of both cations. High-resolution optical fragmentation spectra reveal emission bands due to the CH(A^2^Δ→X^2^Π_r_; B^2^Σ^+^→X^2^Π_r_; C^2^Σ^+^→X^2^Π_r_) and CN(B^2^Σ^+^→X^2^Σ^+^) transitions as well as atomic H and C lines. Their spectral line shapes and qualitative band intensities are examined in detail. The analysis shows that the H_2_^+^ irradiation enhances pyridine ring fragmentation and creates various fragments more pronounced than H^+^ cations. The plausible collisional processes and fragmentation pathways leading to the identified products are discussed and compared with the latest results obtained in cation-induced fragmentation of pyridine.

## 1. Introduction

Ion–molecule interactions lead to the chemical transformation of simple and complex compounds in the atmospheres of Earth, planets, and the interstellar medium [1,2,3,4,5,6,7]. Some collisional reactions are pertinent to combustion processes [8,9]. Luminescence produced under ion beam bombardment is utilized to gain information on defect and impurity sites for insulating materials and semiconductors [10,11,12]. Focused ion beams are employed for etching, deposition, and direct-write lithography and fabrication technology [13,14] and for sample imaging in ion microscopes [15,16,17]. However, the most intense studies are performed to elucidate the ion-induced alterations to structure blocks of living cells, mainly the DNA/RNA molecules [18,19,20,21,22] and their analogs [23,24,25,26,27,28,29,30,31,32]. The reason is that the ionic beams are effectively applied in hadrontherapy to irradiate the cancerous cells [33,34,35]. Moreover, such studies enable predicting the risk of astronauts’ exposure to cosmic rays and the solar wind during eventual human missions to Mars and other planets [36,37,38].

The interaction of ions with biological molecules in the gas phase leads to ionization, excitation, charge transfer, complex formation, and dissociation processes that produce neutral and ionic products [18,19,20,21,22,23,24,25,26,27,28,29,30,31,32]. The mass spectrometric methods are developed to probe the charged entities [22,39], as well as to study neutral fragments in high-Rydberg states but without identifying their final states [40,41]. Conversely, excited products can be identified by detecting their emission [42,43]. Therefore, fluorescence spectroscopies have been utilized extensively to search excited neutral fragments in electron and photon-induced excitation, ionization, and fragmentation of biomolecular targets [42,43,44,45,46,47,48,49,50,51,52,53,54,55,56,57,58].

Although fluorescence spectroscopy is a powerful technique to probe the reaction intermediates and fundamental properties of atoms [59,60,61] molecules [43,62,63], and materials [64], it has been rarely used to explore ion-induced neutral dissociation of polyatomic targets [24,25,26,27,28,29,30,31,32,65]. One of such objects is pyridine C5H5N—the simplest six-membered nitrogen-containing heterocycle compound. The pyridine ring is a component of B vitamins [66], NAD and NADP coenzymes [67], and alkaloids produced by living organisms [67]. Pyridine derivatives such as vitamin B3 (niacin) and nucleobases (pyrimidines, purines) have been discovered in carbonaceous chondrites [68,69]. Therefore, C5H5N is the subject of unremitting astrochemical searches [70,71]. It also has many biological, pharmaceutical, and agrochemical applications (see, e.g., [66,72] and references therein).

The ion-induced processes in pyridine molecules in the gas phase have not been extensively studied thus far, despite their fundamental and industrial importance. For instance, the formation of the free NH(A^3^Π) radical via the hydrogen migration has been recently investigated in collisions of pyridine molecules with the H^+^, H_2_^+^, He^+^, He^2+^, and O^+^ cations exploiting the collision-induced luminescence spectroscopy [27,30]. The creation of the excited NH radicals depended on the type of selected projectile and was selectively activated by tuning the collision velocity. In another research, the collisional excitation products and the spectral signatures of collisional mechanisms in the He^+^ + C_5_H_5_N impact system have been identified by analyzing the collision-induced fragmentation spectra measured at 1000 eV [32]. This investigation established traces of the electron capture processes but with no conclusive evidence for other collisional reactions. To the best of our knowledge, cation-induced fragmentation into neutral excited fragments of pyridine has not been further reported in the literature. In particular, no experimental outcomes have been published comprising the H^+^ and H_2_^+^ ions impinged upon pyridine molecules. Nonetheless, we also mention the mass spectrometric investigations of collisional fragmentation to cover every aspect. Fondren et al. [73] have collided pyridine with several ionic projectiles at the 10–22 eV energy range. However, they only determined rate coefficients for the most intense primary dissociative ionization channels. The crossed molecular beams technique was used to explore the elementary reactions of pyridine but with neutral projectiles, i.e., carbon (C; ^3^P_j_) [74] and nitrogen N(^2^D) [75] atoms, both at collision energies below 1 eV. These studies found few rection channels involving isomerization of the initial collision complexes via ring-opening, ring-expansion, and ring-contraction mechanisms.

Therefore, the current study explores the collisional and dissociation processes in the gas phase pyridine molecules initiated by the impact of protons (H^+^) and dihydrogen cations (H_2_^+^). The collision-induced emission spectroscopy has been used to detect the collisional excitation products at the different kinetic energies of both cations. On that basis, we identified the possible reaction channels involved in the fragmentation of pyridine under the H^+^ and H_2_^+^ cations impact. These ions were selected as projectiles for the investigations because of two reasons. They are the simplest and most abundant cations formed in the cosmos [76,77], and the velocities applied here correspond to slow-speed solar wind [76]. Moreover, medical hadrontherapy procedures usually utilize protons to irradiate deep-seated tumors [78,79], but other ionic beams are also expected to be a propitious source of radiation [79,80,81,82,83]. Hence, H^+^ and H_2_^+^ are model projectiles to explore the ion–molecule interactions in cosmic and biological environments, respectively.

## 2. Results and Discussion

### 2.1. Fragmentation Spectra

**Figure 1:**
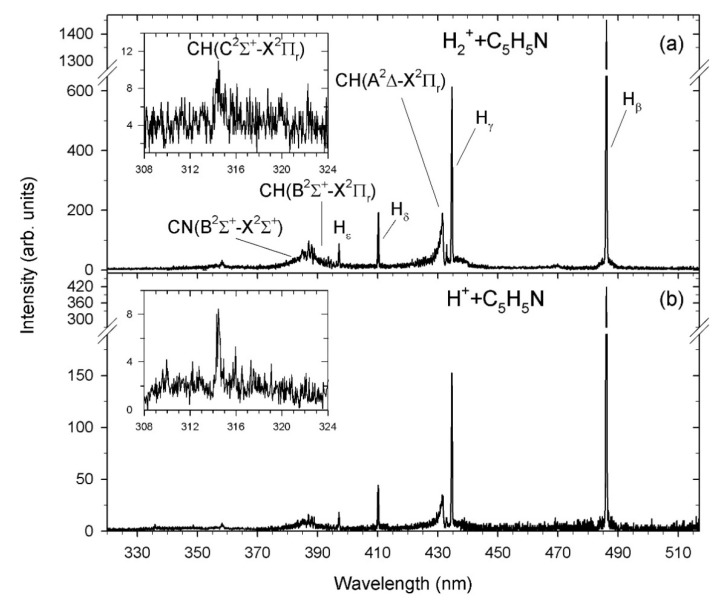
High-resolution optical fragmentation spectra measured for collisions (**a**) H_2_^+^ + pyridine, (**b**) H^+^ + pyridine. The spectra were not corrected for the wavelength dependence of the sensitivity of the optical detection system.

High-resolution optical fragmentation spectra measured for collisions between the H^+^ and H_2_^+^ cations and pyridine molecules are shown in Figure 1. Both spectra show spectral features of the atomic H_β_ to H_ε_ lines of the Balmer series and the molecular CN (B^2^Σ^+^→X^2^Σ^+^) Δν = 0, and CH(A^2^Δ→X^2^Π_r_; B^2^Σ^+^→X^2^Π_r_; C^2^Σ^+^→X^2^Π_r_) Δν = 0 systems. However, the spectra obtained in the H_2_^+^ collisions (Figure 1a) are a bit more complex than the spectra recorded for the H^+^ cations (Figure 1b) and contain a much stronger signal in the case of H_2_^+^ as compared to that obtained from H^+^. Moreover, the relative intensities of the features in both spectra are different. These observations are confirmed by a detailed analysis of the fractional yields of excited fragments (which are further discussed). It is of note that weak emissions of CN(B^2^Σ^+^→X^2^Σ^+^) Δν = 1, NH(A^3^Π→X^3^Σ^−^) Δν = 0 bands and C lines were observed but in spectra measured with much lower resolution.

The present data can directly be compared with our previous pyridine impact studies. In contrast to the present measurement, more severe fragmentation of pyridine irradiated by He^+^ cations was observed [32]. The recorded spectra revealed pronounced emission bands due to the CH(A^2^Δ→X^2^Π_r_; B^2^Σ^+^→X^2^Π_r_; C^2^Σ^+^→X^2^Π_r_) Δν = 0,1, CN(B^2^Σ^+^→X^2^Σ^+^) Δν = 0, ±1, C_2_(d^3^Π_g_ →a^3^Π_u_) Δv = +1, and NH(A^3^Π→X^3^Σ^−^) Δν = 0 transitions, as well as atomic H, He, and C atom lines. Synchrotron radiation studies on pyridine neutral dissociation [56] also showed prominent fluorescence of the diatomic fragments, particularly the CN radical. 

Our luminescence fragmentation spectra may also be compared with the emission spectra measured in collisions involving other heterocyclic biomolecules. In the series of works regarding the cation- [28,31] electron- [44,45], and photon-induced [53,54,55,56,57,58] fragmentation of isoxazole, pyrrole, tetrahydrofuran, pyrimidine, five- and six-membered heterocyclic molecules, we observed complex fragmentation spectra suggesting significant disintegration of their rings. The electron-induced fragmentation processes of larger biomolecules also showed similar nature. Electron collisions with adenine [47,50], cytosine [48], thymine [49], and glutamine [51,52] molecules prompted their decomposition through various channels, the most apparent of which contained the detachment of hydrogen atoms and diatomic radicals in the excited states with the subsequent fluorescence emission. In contrast to the studies discussed thus far, but in line with the observations made here, the light projectiles (H^+^, H_2_^+^, H_3_^+^, He^2+^) dissociated furan and tetrahydrofuran molecules ineffectively, leading mainly to their dissociation into excited H(*n*) atoms [24,25,26,28,31]. 

### 2.2. Theoretical Spectra of CN and CH Radicals

**Figure 2:**
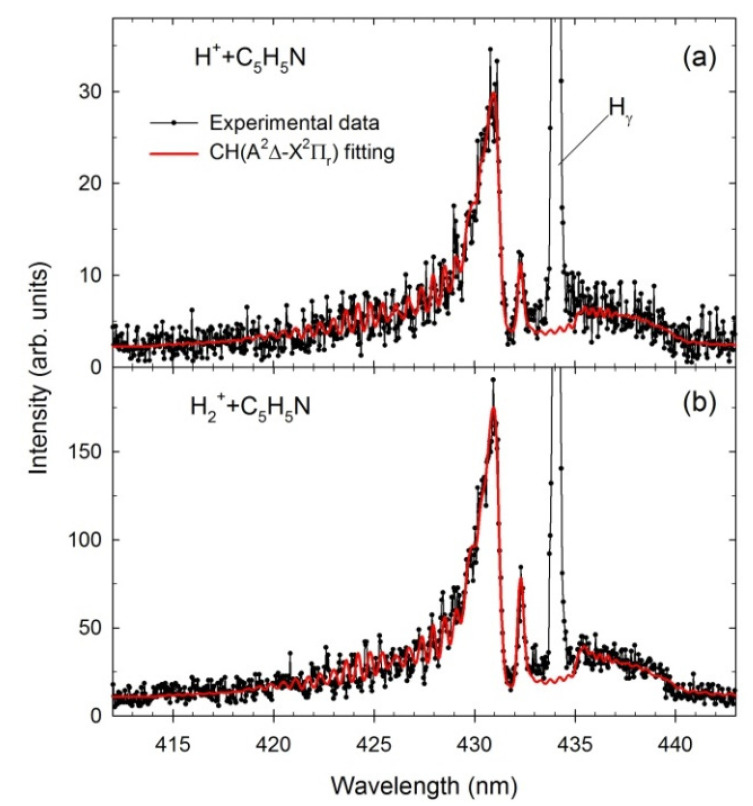
The experimental (black dots) and simulated (red contours) CH (A^2^Δ→X^2^Π_r_) spectra obtained in the (**a**) H^+^ + pyridine (E = 1000 eV), (**b**) H_2_^+^ + pyridine (E = 1000 eV).

**Figure 3:**
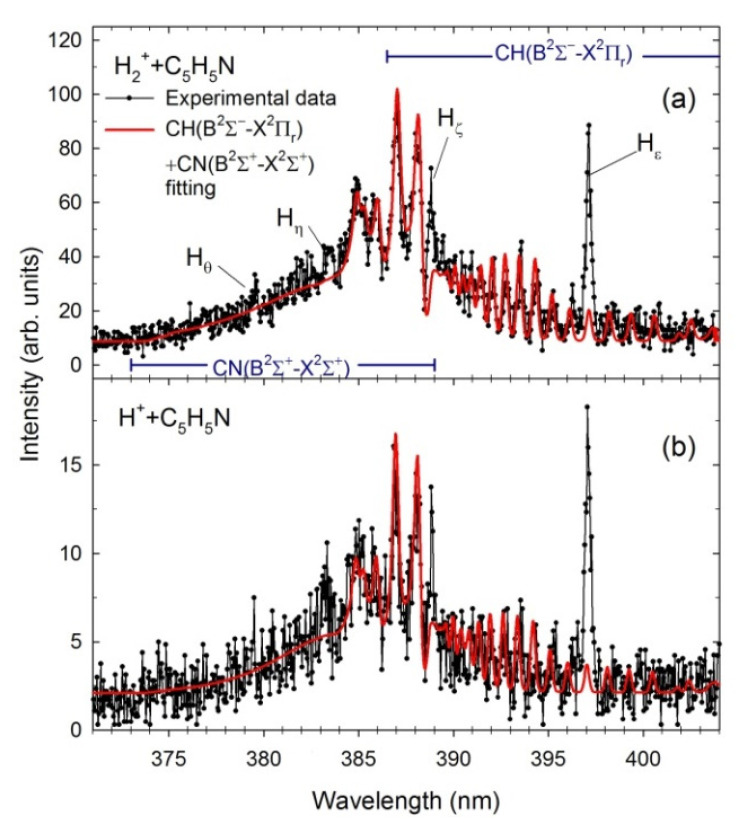
The experimental (black dots) and simulated (red contours) CN(B^2^Σ^+^→X^2^Σ^+^) + CH(B^2^Σ^+^→X^2^Π_r_) spectra obtained in the (**a**) H_2_^+^ + pyridine (E = 1000 eV), (**b**) H^+^ + pyridine (E = 1000 eV).

The theoretical spectra of CN(B^2^Σ^+^→X^2^Σ^+^) Δν = 0 and CH(A^2^Δ→X^2^Π_r_; B^2^Σ^+^→X^2^Π_r_) Δν = 0 molecular bands were next calculated to identify the differences in each impact system. The spectral fittings were achieved by comparing the experimental data with simulations generated using the LIFBASE molecular spectra simulation package [84]. This procedure was initiated in the most intense rovibrational bands of CN and CH because less uncertainty exists in their resultant populations. The line positions and their intensities were computed utilizing the relevant spectroscopic vibrational and rotational constants of the A^2^Δ, B^2^Σ^+^ and X^2^Π_r_ electronic states of CH [85,86,87] as well as B^2^Σ^+^ and X^2^Σ^+^ states of CN [88,89]. Moreover, the rovibrational populations were assumed to be Boltzmann. Figure 2 and Figure 3 show fittings performed for the CH(A^2^Δ→X^2^Π_r_) and CN(B^2^Σ^+^→X^2^Σ^+^) + CH(B^2^Σ^+^→X^2^Π_r_), respectively. The best fits were achieved by employing the Voigt profile for the apparatus function with a resolution Δλ of 0.39 nm (FWHM). The program permits the determination of the characteristic vibrational (*T_v_*) and rotational (*T_R_*) temperatures listed in Table 1. For comparison, the results from He^+^ + C_5_H_5_N collisions [32] are also presented.

The CH emission (414–440 nm) arises from electronic transitions from the A^2^Δ first excited to the X^2^Π_r_ ground electronic level with no change in the vibrational quantum number (Δν = 0). Calculations show that vibrational transitions at this excited electronic level generate characteristic spectral emission bands, with the strongest being related to the (0,0) band. The asymmetric peak at 431 nm is produced by the overlapping Q branches of the (0,0) and (1,1) vibrational transitions. Its low wavelength shoulder consists of R branches, while the structure above 433 nm is built by the P branches. The narrow peak at 432.5 nm was appointed as the head of the Q branch of the (2,2) vibrational transition. The spectra measured for various cations have the same primary components, but they differ slightly in shape due to different temperatures. Both *T_v_* and *T_R_* increase with increasing cation mass (see Table 1). As a result, the small bump at 433.5 nm becomes visible at the CH(A^2^Δ→X^2^Π_r_) Δν = 0 spectra obtained for H_2_^+^ (Figure 2b) and He^+^ (see Figure 1 in [32]) collisions. Calculations show that this feature is related to the head of the Q branch of the (3,3) vibrational transition, which only appears at higher *T_v_.*

Because CN(B^2^Σ^+^→X^2^Σ^+^) band overlaps with the CH(B^2^Σ^+^→X^2^Π_r_), the sum of their simulated contours is shown in Figure 3 for both impact systems. Figure 3 also exhibits the H_ε_ to H_θ_ lines of the Balmer series arising from higher excitations of H(*n*), *n* = 7–10. The 370–389 nm spectral region displays luminescence of the B^2^Σ^+^→X^2^Σ^+^ rovibrational bands of the CN molecule. This emission results from electronic transitions with no change in the vibrational quantum number (Δν = 0). Calculations show that the band heads of the *P* branches of the (0,0), (1,1), and (2,2) transitions produce the peaks at 388.3, 387.1, and 386.0 nm, respectively. The relative peak amplitudes of the *P* heads on the high wavelength side depend on the vibrational temperature. The rotational temperature determines the shape of a tail towards a lower wavelength corresponding to transitions between higher rotational levels. In this case, both temperatures increase as the mass of the cation rises. In addition, *T_v_* temperatures are on average twice as high as *T_R_* ones. Note that the *T_v_* vibrational temperatures used in the fittings to CN spectra measured in the photodissociation of pyridine [56] were slightly lower but gradually increased from 5000 to 7200 K in the 16–27 eV photon energy range. The *T_R_* rotational temperatures raised subsequently from 3000 to 4300 K at those energies [56].

The 386.5–409.0 nm spectral region reveals luminescence of the CH molecule’s second B^2^Σ^+^→X^2^Π_r_ rovibrational band. Its structure consists of the rotational lines of the (0,0) and (1,1) vibrational transitions: the (0,0) rovibrational lines spread over the 386.5–402.5 nm wavelength range while the (1,1) band appears at the 402–409 nm. In addition, the *R* branch’s band head of the (0,0) transition of the CH(B^2^Σ^+^→X^2^Π_r_) overlaps with the (1,1) vibrational band of the CN. In addition, here both temperatures slightly increase as the mass of the cation rises. However, they are lower than those obtained for previous systems. In addition, *T_v_* temperatures are almost on the same level as *T_R_* ones contrarily to fittings to CH(A^2^Δ→X^2^Π_r_) and CN(B^2^Σ^+^→X^2^Σ^+^) bands.

The above analysis shows that the diatomic products yielded in collisions are highly energized. This observation implies that the fragmentation process is vigorous, releasing a large amount of energy. Recent experiments concerning the production of CH(A^2^Δ) and CN(B^2^Σ^+^) radicals utilizing intense femtosecond near-IR laser pulses [90,91] showed the interlink between the nature of molecular dissociation and the level of *T_v_* and *T_R_* temperatures and their respective energies. High temperatures, particularly of both radicals’ rotational energies, indicated the sequential character of the parent molecules dissociation in agreement with a so-called impulsive model [92]. High values of these temperatures obtained here may thus suggest that collisional interaction is a first step that triggers a cascade of further elementary reactions. Molecular fragments can only be formed in complex fragmentation of pyridine, which proceeds via ring-opening reactions [93]. The effectiveness of the momentum transfer of the breaking bonds into the internal degrees of freedom of the fragments should then depend on the geometry of the open ring structures [94]. The efficiency of rotational excitation of a particular molecular fragment depends on the reduced mass of reactants and the distance of the impulsive recoil from its center of mass [94]. This decomposition pulse additionally may provide unevenly distributed fragments around the center of mass. Using the velocity mapping ion imaging, Pei & Farrar [95] have recently studied the charge transfer reaction between C^+^ and NH_3_ and observed such an asymmetry of the product flux distributions in relation to the center of mass direction. Their kinetic energy distributions additionally suggested the efficient formation of a range of product’s vibrational states [95], thus lending support to the impulsive model scenario.

### 2.3. The H(n) Intensity Ratios

In the next step, the characteristics of hydrogen Balmer series intensities were examined. As seen in Figure 1, the recorded spectra exhibit the hydrogen atomic lines, whose intensities decrease quickly. In quantum mechanics, the H(*n*) intensities are proportional to the principal quantum number *n*, according to the *I*~*n^−3^* relationship [96].

**Figure 4:**
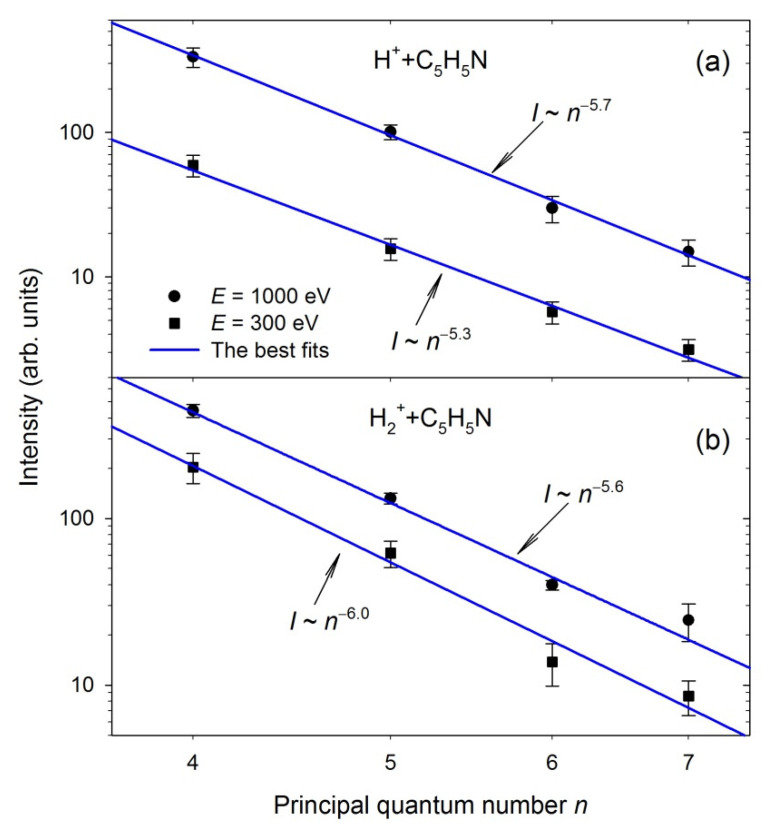
Examples of the log–log plots representing the Balmer line intensities as a function of the principal quantum numbers in the collisions of (**a**) H^+^ + pyridine, (**b**) H_2_^+^ + pyridine. The solid lines show the best fits to the experimental points.

**Figure 5:**
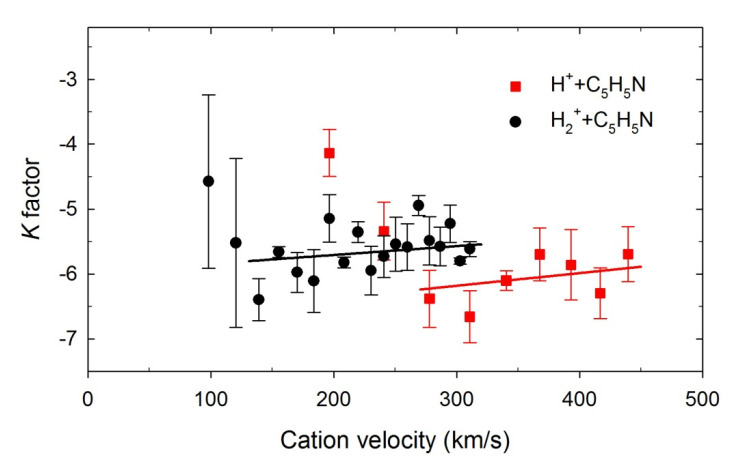
The *K* factors as a function of incident cation velocity. The solid lines represent overall trends at higher velocities.

In Figure 4, the H(*n*), *n* = 4–7, intensities corresponding to H_β_–H_ε_ Balmer lines measured at two example cations energies are plotted in the log-log scale as a function of the principal quantum number *n*. These dependencies were fitted for each projectile energy using a least-squares-fitting procedure by an *n^K^* exponential function, where *K* was an adjustable parameter. Accordingly, straight lines were obtained, as shown in Figure 4, and the *K* values were determined. In Figure 5, the calculated *K* factors were drawn as a function of each cation’s velocity. Both curves show a similar trend. They are generally two times lower than the expected theoretical value of (−3). The *K* factors decrease from about (−4) down to their minimal values at the lowest velocities. Then, they slowly, monotonically, increase with rising velocity and reach the maximum values of an order of (−5.7).

The differences in the *K* values may suggest that diverse processes produce and populate the H(*n*) atoms. The *K* factors near (−3) evince the production of the H(*n*) atoms in their substates populated according to their statistical weights. This *K* value can be related to H(*n*) hydrogen production via dissociative processes. Recent studies of electron- [44] and photon-induced [53,54] dissociation of heterocyclic molecules have established that the H(*n*) intensities follow the *n*^−3^ relation. In addition, the H(*n*) intensities of hydrogen atoms obtained in C^+^/O^+^ + THF collisions fulfill this rule [28]. Values of *K* factors determined from our measurements are lower than (−3), which means that excited states in hydrogen leading to Balmer emission produced during H^+^/H_2_^+^ + pyridine collisions were not populated equally. In particular, higher-lying excited states of H(*n*) were strongly depopulated.

Poorly populated Hydrogen atoms can only be formed from the beam of neutralized H^+^/H_2_^+^ projectiles [25,26,28], thus suggesting that the charge transfer process may be responsible for creating the excited hydrogen atoms in that way. The movement of H^+^/H_2_^+^ projectiles in the Earth’s magnetic field may generate the electric field due to the space charge phenomenon. It is well-known that electric fields via Stark mixing may influence the branching ratios and lifetimes of specific states. High electric field strengths alter the populations [97,98,99,100], but the remnant electric field existing in the collision cell can also modify the wave functions of the excited states [101]. The electric field strengths higher than 0.22 V/cm were estimated by us previously for H^+^ projectiles [26]. Such field strengths are high enough to induce the Stark mixing capable of depopulation of the *n* = 6, 7, 8, …, levels, thus leading to the observed decrease of *K* values. Note that the velocity of diffusive pyridine molecular beam and, consequently, hydrogens produced from its fragmentation were too low to generate the electric field strengths capable of Stark mixing [102].

### 2.4. Emission Yields

**Figure 6:**
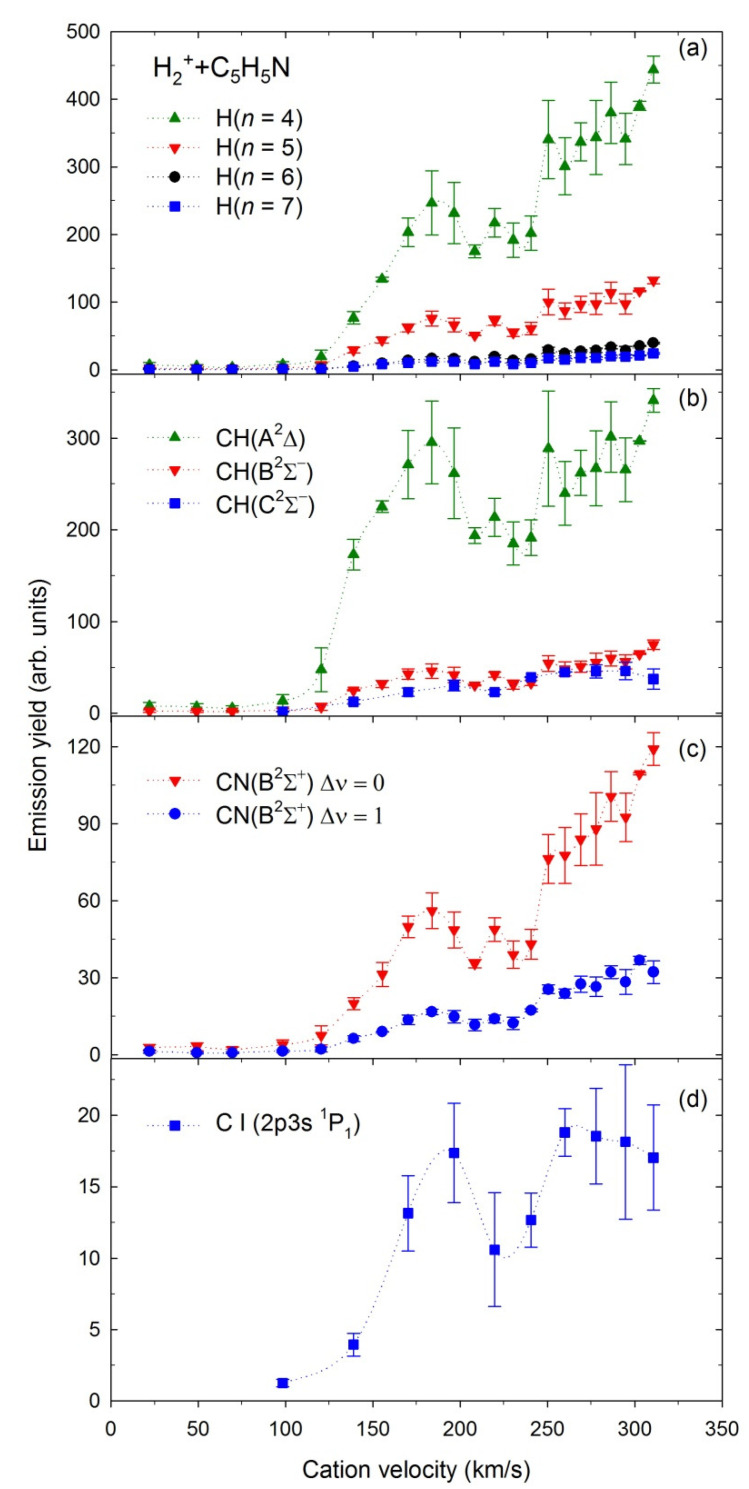
Emission yields of the excited fragments obtained in collisions of H_2_^+^ with pyridine.

**Figure 7:**
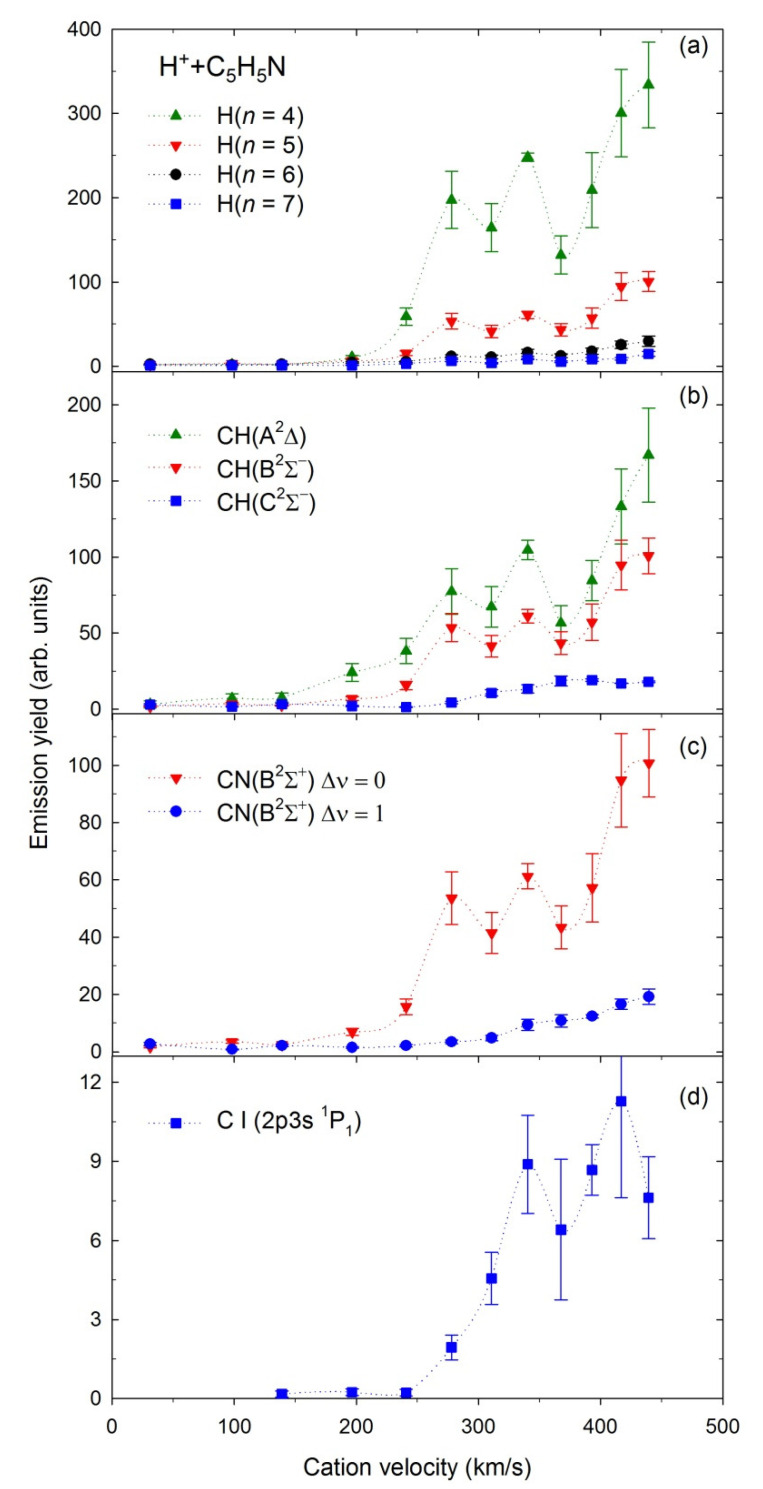
Emission yields of the excited fragments obtained in collisions of H^+^ with pyridine.

Emission yields (representing relative emission cross-sections, σ—for more details, see Section 3) for production of the H(*n*), *n* = 4–7, CH(A^2^Δ), CH(B^2^Σ^–^), CH(C^2^Σ^+^), CN(B^2^Σ^+^), and the C(2p3s ^1^P_1_) excited fragments in collisions with the H^+^ and H_2_^+^ cations depicted as functions of the projectiles’ velocities are shown in Figure 6 and Figure 7, respectively.

The experimental uncertainties are the mean standard deviations obtained from independent measurements performed at each impact velocity. The ion beam energy spread and thermal motion of the target determine the uncertainty of collision velocity, which was estimated to be less than 3.5%.

The measurements with H^+^ were performed in the 5–1000 eV energy range with 100 eV step above 100 eV. These energies correspond to velocities of 31–440 km/s. The emission yields obtained for the above fragments rise gradually in the presented velocity range. However, some fluctuations are observed for velocities of 250–350 km/s.

The measurements with H_2_^+^ were carried out in the 5–1000 eV energy range (velocity range 22–311 km/s) with 50 eV step above 50 eV. The *σ* curves recorded for H_2_^+^ have different contours that depend on the velocity. They rise rapidly above 125 km/s and show resonance-like maxima peaking at 175 km/s and having a half-width of ~80 km/s. Above 225 km/s, almost each emission yield rises monotonically. However, the H(*n*) and CN(B^2^Σ^+^) curves at the maximum reach almost twice the values of the resonance-like peaks. In contrast, CH(A^2^Δ), CH(B^2^Σ^−^), CH(C^2^Σ^+^) emission yields do not increase as promptly. The C(2p3s ^1^P_1_) emission yield reaches a plateau above 250 km/s.

The average relative abundances (*RA*) given as the fractional yields of individual excited fragments to the total yield of all identified emitting products (recorded in 190–520 nm wavelength) and averaged over the whole cation energy range have been calculated to determine the trends in different collisional systems. Table 2 displays the obtained results of H^+^/H_2_^+^ + C_5_H_5_N. The results of He^+^ + C_5_H_5_N [32] and H^+^/H_2_^+^/H_3_^+^ + C_4_H_8_O [24,25,26] collisions are also presented in Table 2 for comparison.

Six observations are noted from the average relative abundances assembled in Table 2: (i) production of H(*n*) atoms decreases linearly with an increase of the hydrogen cation mass similarly for pyridine (C_5_H_5_N) and tetrahydrofuran (C_4_H_8_O). (ii) In contrast, the formation of CH excited radicals increases with increasing the hydrogen cation mass correspondingly for pyridine and tetrahydrofuran. (iii) The creation of other emitting products (i.e., CN, NH, C) is generally at the same level for both H^+^ + C_5_H_5_N and H_2_^+^ + C_5_H_5_N impact systems. (iv) Collisions with He^+^ cations dramatically diminish the generation of H(*n*) and increase the production of other products, thus enhancing pyridine ring fragmentation significantly. (v) The emission of NH(A^3^Π) radicals indicates the hydrogen atom relocation prior to the cation-induced fragmentation (see [27] for details). Such isomerization leading to NH(A^3^Π) formation occurs at a comparable level in all collisional systems. (vi) Observation of He emission lines is direct evidence of single electron transfer from pyridine to He^+^ projectiles before the fragmentation (discussed in detail in [32]). However, it is not a significant collisional process (*RA* equal to 1.6%). A rationale for these observations will be established further below.

### 2.5. Elucidation of Collisional and Fragmentation Processes

Recent experimental [18,19,24,25,26,27,28,32], and theoretical [31,103] investigations have shown that five collisional processes occur prior to fragmentation:

(i) Charge-transfer (CT) that ensues via an electron relocation from C_5_H_5_N to the H^+^/H_2_^+^ cations, followed by fragmentation of C_5_H_5_N^+^ parent cation of pyridine. This reaction is usually exothermic [104], which enables the transfer of a significant amount of energy into internal degrees of freedom of molecular products. CT reaction occurs readily at relatively long projectile−target distances [18,19,31,103].
(ii) Dissociative excitation (DE) involves excitation and further fragmentation of pyridine molecules.
(iii) Dissociative ionization (DI) represents direct ionization of the pyridine molecule accompanied by excitation and fragmentation of the pyridine cation. Alvarado et al. [105], in their investigations on interactions of keV H^+^ and He^q+^ with isolated deoxyribose molecules, assumed that the creation of small fragments is associated with violent close collisions involving mainly direct ionization accompanied by electronic and vibrational excitation.
(iv) The fourth reaction is a transient cation–molecule complex formation (TC) owing to an ion−dipole interaction [106]. The constituent units interact electrostatically due to the attractive force between the charge of the H_2_^+^ ions and the permanent dipole moment (2.21 D [107]) of pyridine. Akin to the charge transfer reaction, the complex formation occurs at a relatively long projectile−target distance. The ab initio quantum chemical calculations of the collisions of He^+^/He^2+^ cations with furan [31] have recently shown significant changes of the wave functions leading to avoided crossings at the potential energy curves around R = 1.5–2.0 Å. Physically this means that electronic clouds of the target and the projectile start overlapping at this length, thus merging both reactants into [He−C_4_H_4_O]^+/2+^ temporary cluster. Note that the CT mechanism also occurs via avoided crossings and, in principle, can also be regarded as the formation of a quasimolecular complex [31].
(v) The fifth mechanism that we can ascertain is a direct dissociative excitation of an H_2_^+^ projectile (DP) since the H_2_^+^ is a molecule that can be decomposed during collisions.

As suggested in the Section 2.2, each collisional interaction may prompt sequential fragmentation leading to observed products. In this regard, we calculated the simple thermochemical values of the lowest threshold energies (*E_TH_*) for reactions producing the most intense excited fragments in the collisions of H^+^/H_2_^+^ with pyridine. These estimations (listed in Table 3) were performed utilizing the dissociation, ionization, and excitation energies from papers [53,55,56,108,109,110,111,112,113,114]. Note that five carbons build the skeleton of the pyridine molecule, but their detachment from the ring requires complex, multistep, and energy-consuming decomposition that is difficult to decipher. Moreover, the NH(A^3^Π) radicals appear in the isomerization of pyridine via the H atom migration, as has been described by us in the preceding papers [27,58]. Therefore, dissociation channels leading to excited C and NH and multistep processes leading to H, CH, and CN species are not considered in Table 3.

As seen in Table 3, capturing an electron by the H+ and H_2_^+^ projectiles from pyridine is an exothermic reaction. For that reason, it is energetically the most privileged process. H(n = 4), CH(A2Δ), and CN(B2Σ+) all have the lowest appearance energies after the charge transfer reaction. We did not attempt to measure accurate collision energy thresholds for the observed emissions. However, traces of CH, CN and even H emissions should be observed in the spectra measured at the lowest energies if this process occurs. In-depth examination of the luminescence spectrum measured for 5 eV H_2_^+^ cations showed the existence of excited CH, CN emission bands, and Hβ line, thus corroborating our supposition. Their emission deteriorated in the spectrum measured at an energy of 25 eV. These observations agree with recent ab initio quantum chemical calculations of the collisions of protons with the pyrimidine nucleobases, uracil, and thymine [103]. The calculations showed avoided crossings around R = 2.1 Å between the entry channel and the charge transfer state corresponding to a double excitation at the potential energy curves of the different molecular states involved in H^+^ + uracil and H^+^ + thymine collisional systems [103]. These anti-crossings induced efficient electron charge transfer because this process is prompted chiefly by the nonadiabatic interactions between the adiabatic potential curves at their nearest points [103]. Thus, reactants do not have to be close together to feel the attraction, and the CT reaction can take place even at low energies. The corresponding charge transfer cross-sections for both pyrimidine nucleobases were the highest below 10 eV (~10−3 × 10−16 cm^2^) and decreased slowly (to about 10−4 × 10−16 cm^2^) with increasing collision energy [103]. Suppose a similar situation occurs in the present collisions. In that case, the production of exited products at 5 eV results from the nonadiabatic interactions between the adiabatic potential curves, and the drop of the CT cross-sections can explain the decrease in the intensity of the emitting products. However, a final description of this process can only be supported by theory, and thus the quantum chemical calculations are required for the present impact system.

For proton collisions, a situation is a little bit puzzling. The spectra measured with H+ cations at the 5, 50, and 100 eV energies revealed no exciting products. However, high values of H(n) relative abundances (see Table 2) suggest that this process occurs here and dominates the H^+^ + pyridine collisions. The absence of emission at these spectra can perhaps be rationalized by remarking that the typical H+ cation beam current was several times lower than H2+ ionic flux. Thus, it was too low for detecting the CT reaction signal. If we increased the recording time significantly, we could likely observe traces of this reaction. Nevertheless, the latest experimental [26,28,105], and theoretical [103] investigations of fragmentation and collision-induced ionization of biomolecules established that capturing electrons from the targets to protons is the primary impact mechanism. A significant portion of the energy is then embedded into the neutralized H+ cations [105], thus giving excited hydrogen atoms. The excited hydrogen atoms can be generated via the CT reaction either by excitation of neutralized H^+^ (reaction no. 2) or by detaching hydrogen from the pyridine parent cation. The latter process can occur in two general ways. Hydrogen can be detached from a closed ring of pyridine (no. 3) or the open ring structure (no. 4). These likely reactions differ only by 1.11 eV, but they still require 4.78 eV more energy than reaction no.2.

As suggested in the Section 2.3, substantial depopulation of H(*n*) higher-lying excited states indicates that hydrogens were produced from cations via charge transfer reaction from pyridine to projectiles. However, in the H_2_^+^ + pyridine collisions, the H2 must first decompose (see reaction no. 19). This process increases the reaction energy. Therefore, detachment of hydrogens from pyridine after electron transfer reaction requires only 2.13 eV more energy (reaction no. 20) and can efficiently compete with the fragmentation of H_2_. In addition, the production of other fragments becomes more competitive. In general, this can lead to decreased overall production of excited hydrogens. The relative yield of hydrogen species decreased from about 61.4 to 45.9%, going from the H^+^ + C_5_H_5_N to H_2_^+^ + C_5_H_5_N impact system (Table 2).

Some fraction of excited hydrogens may come from the direct dissociation of excited H_2_^+^ projectiles (see reaction no. 22). However, direct dissociative excitation of an H_2_^+^ requires 4.82 eV more energy even than the energetic CT reaction (no. 21), and we exclude this decomposition channel.

For both cations, dissociative excitation and dissociative ionization processes require several electronvolts more energy than the charge transfer mechanisms, thus becoming less competitive processes regarding the CT reaction. Recent ion-induced fragmentation investigations corroborate these observations. Alvarado et al. [105] found that the electron transfer reaction releases most of the energy on the projectile, particularly the H^+^ cation. This result is also consistent with ion-impact studies of Mayer and co-workers [65], who examined collisions of the H_2_^+^/He^+^ with methane, acetylene, benzene, and naphthalene and the curve-crossing mechanism for collisional activation. Their calculations of adiabatic potential energy curves for the ground and excites states of the collisional clusters excluded the possibility of effective dissociative excitation of the target molecules after the H_2_^+^ cation bombardment [65]. In addition, the most recent studies on H_2_^+^ collisions with tetrahydrofuran [25] suggested increased H(*n*) production via CT reaction that most likely dominated any other mechanism.

Note that electron transfer occurs even in systems with resonant electron capture energetically prohibited. For instance, spectroscopic signatures of CT reactions have been recently observed in the collisions of He^+^ with furan [31]. It was surprising because the energy levels of He^+^ and furan do not coincide at all, which significantly hinders the resonant transfer of electrons from furan’s highest occupied molecular orbital to He^+^. A similar situation could occur in the case of He^+^ + pyridine collisions. The energy levels of interacting entities did not have similar energies, which hampered the CT reaction. Therefore, a single electron transfer from pyridine to He^+^ projectiles was observed as an insignificant collisional process (see Table 2).

The binding of the projectile and the target in a collision complex is complicated, and only quantum-chemical calculations can describe this mechanism. Therefore, temporary complex formation was omitted in our simple estimations, although in many gas-phase phenomena, such complexes are important intermediate species, often leading to unusual processes [27,28,31,106,115,116]. Nevertheless, some hints indicate the formation of such clusters in the present collisions. A high level of quantum chemistry calculations [31] showed that the formation of the [He–C_4_H_4_O]^+/2+^ temporary clusters before decomposition was responsible for the appearance of the narrow resonances in the relative cross-section curves at lower velocities of He^+^. Our emission yields measured for H_2_^+^ demonstrate clear resonance-like maxima (Figure 6), thus indicating the formation of the [H_2_–C_5_H_5_N]^+^ ion-neutral molecule temporary complexes prior to dissociation. Such complexes may arise because the pyridine molecule has the π-electron system and the nonbonding electron pair of nitrogen heteroatom as attractive sites for molecular interactions [107]. Theoretical calculations suggested that the electrostatic potential on the nitrogen atom can bound stable van der Waals σ_N_-type complexes rather than the π-electron ones [107,117,118]. However, calculations concerning interactions of pyridine with various hydrides suggested that interactions in the σ_N_-type complexes are predominantly electrostatic, while the dispersion connects the π-type clusters [119]. Moreover, ab initio calculations identified propeller-shaped isomers of (Benzene·Pyridine)^+^ covalently bonded via a C–N bond [120], which has been recently observed experimentally in pyridine clusters [121]. These works show that pyridine can create complexes, which would probably also be formed during present collisions.

Note that some fluctuations having the resonance-like character appear to be at emission yields measured for H^+^ (see Figure 7), but their origin is mysterious at the moment. The only explanation could be the formation of temporary collision complexes [27,28]. The protonated pyridine (C_5_H_5_NH^+^) where the protonation site is at the nitrogen atom has been observed employing cold ion infrared action spectroscopy [122]. A high proton affinity (9.7 eV) of pyridine [121] may be a factor in forming such a complex. However, thus far, there has been no direct spectroscopic evidence confirming the occurrence of the complexes in the collisions with H^+^ projectiles [26,27,28,30].

Calculations of adiabatic potential energy curves for the ground and excited states of the [He + naphthalene]^+^ collisional cluster found crossing points between them, thus suggesting increased probability for collisions with the formation of excited states of the naphthalene molecule [65]. After the breakup of the cluster, its excitation can be transferred to excited states of the reactants, yielding enhanced overall production of emitting products. This result may explain the enriched luminescence fragmentation spectra observed by us and Mayer’s group [65] when He^+^ cations were used as projectiles.

Notwithstanding the arguments we have presented above, all emitters excluding the hydrogen atoms can only come from the dissociation of pyridine. The earliest works considered mainly dissociation of pyridine into the ground state products. Such products were observed in pyrolysis [123,124,125] and laser-induced photodissociation [93,126] of pyridine. Ab initio density-functional calculations of geometries and energies of various pyridine isomers showed that their fragmentation occurs in the ground electronic state via ring-opening reactions involving extensive isomerization [93]. Zhong et al. [127] indicated a role of conical intersections in driving these ground-state processes. However, an internal conversion would prevent dissociation due to the enhanced density of higher-lying excited states (likely populated in the present experiment). In contrast, Lobastov et al. [128] traced the ring-opening mechanism in pyridine excited to the singlet S_1_ state using the ultrafast electron diffraction technique. They observed that the C–N bond scission caused the production of a hot ring-opened diradical structure characterized by increased vibrational internal energy. The photodissociation studies of pyridine in valence regime [56,57,58] enabled an insight into the fragmentation mechanism, including the excited states. They showed that dissociation of pyridine occurred via its initial excitation into the higher-lying superexcited Rydberg states, followed by rearrangement and isomerization [56,57,58]. Then, these states decayed into two, three-, or even four-fragment channels producing vibrationally and rotationally excited fragments. These fragments were usually cleaved from terminating parts of the molecular chain of open-ring structures [56,58].

Considering the above discussion, we propose a decomposition mechanism that could produce exciting fragments other than H(*n*). Both H^+^ and H_2_^+^ cations’ kinetic energies are high enough to generate superexcited pyridine molecules via one of the discussed collisional reactions (most likely CT or TC ones). Such superexcitation likely prompts a cascade of further elementary reactions, including their geometrical structures rearrangement, either via isomerization or directly through bond breaking and generation of ring-opening structures, similar to those found in the ab initio calculations for the ground states. Apart from electronic excitation, these structures can carry an amount of vibrational and rotational energy. This energy must be transferred to the reaction products because it cannot be dissipated to surrounding solvent molecules under single collision conditions in gas-phase experiments. The easiest way to obtain a particular fragment is to detach it from the outermost parts of the open-ring structures, which usually requires a small number of bonds to rupture. In such a scenario, the bonds break far from the center of the mass of the open-ring structure, and the arising impulsive recoil will additionally increase the rotational energy of the escaping fragment.

## 3. Materials and Methods

The experiments were performed at the University of Gdansk using collision-induced emission spectroscopy (CIES) described in detail in [26,129,130]. The up-to-date picture of the spectrometer and method of measuring was described in [27,28]. Therefore, only a sketch is given here, combined with data pertinent to pyridine.

The setup incorporated individual vacuum chambers intended for the source of cations, the magnetic mass selector, the collision cell, and the optical detection system. The first chamber contained the Colutron type ionic source cooled with liquid nitrogen. A hot cathode discharge produced protons and dihydrogen cations from the H_2_ gas under 50 and 100 Pa pressure. The cations occurring in the plasma were attracted by a 1000 V electric potential and moved to a 60° magnetic mass selector. The cations of a required m/q ratio were filtered from that beam in a mass selector. Then they were decelerated to appropriate velocity/energy using immersion lenses and then transferred to the collision cell. The measurements for the H^+^ + pyridine collisions were performed for the 5–1000 eV projectiles energies, which covered the velocity regime of 30–440 km/s. The research using H_2_^+^ cations was carried out in the 5–1000 eV energy range corresponding to the 22–311 km/s velocity range. The reached cation beam current in the collision region was about 0.21 nA at 5 eV and rose to 0.97 nA at 1000 eV for protons and 1.0 nA at 5 eV and 2.6 nA at 1000 eV for H_2_^+^. For the proper normalization of photon signals, the ionic beam currents were monitored simultaneously with the measurements of photon emission rates. In the collision cell, each cation beam traversed pyridine vapors. As a result of collisions, exciting products were generated. We estimated that the lifetimes of these emitting products were short enough to emit all luminescence before escaping from the collision zone. The optical emission was then dispersed by McPherson 218 spectrograph and acquired by a 1024 channel “Mepsicron” multi-channel photon detector mounted in the detection chamber.

The collection of experimental data was performed in two spectrometer operating modes. First, the 1200 l/mm grating was utilized to measure the high-resolution spectra Δλ of 0.4 nm (FWHM) at a fixed ion beam energy of 1000 eV. Such a measurement allowed assigning the spectral features accurately. However, the products’ intensities were weak, and long acquisition times were required. Therefore, we employed a 300 l/mm grating in the second operating mode to collect more light and measure emission yields. The spectra were characterized by a lower optical resolution (Δλ of 2.5 nm), still sufficient to separate luminescence features. The detection efficiency curve of the optical system working on the 300 l/mm grating was previously determined using a standard monochromatized light source [131]. The emission yields were measured as follows: the luminescence signal of the selected spectral line or molecular band was monitored as the incident cation energy was scanned over the entire energy range. The measurement at single energy was repeated several times to accomplish good statistics. Each spectrum was corrected for the wavelength dependence of the optical system sensitivity. The intensity of a particular emission line or band was obtained by integrating over the peak/band area and normalizing to collecting time, cation beam current, and pressure. These emission yields provided us with relative emission cross-sections (*σ*), i.e., formation probabilities of the observed products. In this paper, we presented emission yields and *K* factors as a function of the velocity of individual cations, which allowed direct comparison between different impact systems.

The liquid pyridine (C_5_H_5_N) was purchased from Sigma Aldrich, Poznań, Poland. The declared purity of the sample was 99.8% [132]. It was outgassed several times through freeze-pump-thaw cycles until the residual gases evaporated. The measurements were performed at room temperature. We did not heat the sample because pyridine is volatile—it has 10 mmHg vapor pressure at 13.2 °C [132]. The dependence of the luminescence intensity as a function of the pyridine vapor pressure was checked in the range up to 30 mTorr. In this pressure range, it was linear, suggesting measurement in a single impact regime. Nevertheless, the pressure of the pyridine vapors was kept at 15 mTorr to ensure that secondary collision mechanisms did not play any role. During the measurements, the pressure of the pyridine vapors was controlled with the Barocel capacitance manometer.

## 4. Summary

H^+^/H_2_^+^-induced dissociation of pyridine into excited neutral fragments has been investigated experimentally in the 5–1000 eV energy range. Collision-induced emission spectroscopy has been employed to measure high-resolution luminescence spectra in the wavelength range from 190 to 520 nm and search for the spectral signatures of collisional mechanisms. The spectra displayed a shared set of excited atomic and diatomic products even at the lowest cations’ energies. Although experiments have been performed in similar conditions, the products had different relative intensities. There was enhanced production of the Balmer series lines, whose intensities decreased with increasing principal quantum number *n* more quickly than predicted by quantum theory. The relative yield of hydrogen species decreased with increased cation mass. Moreover, the collisions with H_2_^+^ yielded vibrationally and rotationally hotter molecular fragments than collisions with H^+^. Detailed analysis of the spectra and the lowest threshold energies indicate that the charge transfer reaction prevailed during collisions. The recorded emission yields revealed significant variations with the change of the projectile velocity. In particular, at lower velocities of H_2_^+^, the relative cross-sections of dissociation products had prominent resonance-like maxima, which were tentatively assigned to the formation of the [H_2_–C_5_H_5_N]^+^ temporary clusters before decomposition. On that basis, the most likely scenario of the course of collision mechanisms and pyridine decomposition has been proposed.

However, a complete picture of collisional processes and their importance during pyridine fragmentation can only be explained in coupling with the theory. Conversely present experimental results may be utilized as initial parameters for benchmarking state-of-the-art theoretical calculations applied in many areas, such as modeling planetary atmospheres or simulating interactions of ionizing radiation with biological tissues and high-tech materials.

## Figures and Tables

**Table 1:** Characteristic vibrational (*T_v_*) and rotational (*T_R_*) temperatures obtained from the fittings. The data for He^+^ + C_5_H_5_N collisions was taken from [32]. The uncertainties were 500 and 200 K for *T_v_* and *T_R_* in all impact systems, respectively.

Transition	H^+^ + C_5_H_5_N		H_2_^+^ + C_5_H_5_N		He^+^ + C_5_H_5_N	
	*T*_v_ [K]	*T*_R_ [K]	*T*_v_ [K]	*T*_R_ [K]	*T*_v_ [K]	*T*_R_ [K]
CH(A^2^Δ→X^2^Π_r_) Δν = 0	7900	4100	9500	4200	10,000	4800
CH(B^2^Σ^−^→ X^2^Π_r_) Δν = 0	3000	3200	3800	3500	3900	3500
CN(B^2^Σ^+^→ X^2^Σ^+^) Δν = 0	9000	4500	9500	5500	13,500	6000

**Table 2:** The average relative abundances (*RA*) of excited fragments. The results of He^+^ + C_5_H_5_N were taken from [32], while the results for H^+^, H_2_^+^, and H_3_^+^ collisions with tetrahydrofuran were adopted from [24,25,26], respectively. The NH(A^3^Π) data from [27] were also used. The numbers in brackets represent the calculated uncertainties.

Fragment	*RA* (%)											
	H^+^ + C_5_H_5_N		H_2_^+^ + C_5_H_5_N		He^+^ + C_5_H_5_N ^†^		H^+^ + C_4_H_8_O		H_2_^+^ + C_4_H_8_O		H_3_^+^ + C_4_H_8_O	
H_β_	43.6 (6.0)	61.4	32.0 (3.7)	45.9	5.1 (0.6)	10.8	67.4 (5.2)	88.8	57.4 (4.5)	76.2	51.1 (3.0)	67.3
H_γ_	12.5 (2.0)		9.6 (1.1)		3.6 (0.5)		15.0 (2.0)		12.5 (2.2)		11.2 (1.8)	
H_δ_	3.6 (0.7)		2.6 (0.3)		2.1 (0.4)		4.7 (1.0)		4.7 (0.9)		3.5 (0.7)	
H_ε_	1.6 (0.3)		1.7 (0.2)		-		1.7 (0.5)		1.6 (0.5)		1.5 (0.4)	
CH(A^2^Δ→X^2^Π_r_) Δν = 0	19.9 (3.4)	26.9	30.6 (3.7)	40.4	28.2 (1.4)	42.2	8.8 (2.0)	11.2	18.9 (1.9)	23.8	26.6 (2.3)	32.7
CH(B^2^Σ^−^→X^2^Π_r_) Δν = 0	4.1 (0.6)		5.6 (0.7)		11.7 (0.9)		2.4 (0.9)		4.9 (1.0)		6.1 (0.8)	
CH(C^2^Σ^+^→X^2^Π_r_) Δν = 0	2.9 (0.4)		4.2 (0.6)		2.3 (0.5)		-		-		-	
CN(B^2^Σ^+^→ X^2^Σ^+^) Δν = 0	7.1 (1.3)	9.2	7.9 (0.9)	10.4	23.4 (1.3)	27.8	-	-	-	-	-	-
CN(B^2^Σ^+^→ X^2^Σ^+^) Δν = 1	2.1 (0.3)		2.5 (0.3)		4.4 (0.8)		-		-		-	
NH(A^3^Π→X^3^Σ^−^) Δν = 0	1.0 (0.2)	1.0	1.5 (0.4)	1.5	1.0 (0.4)	1.0	-	-	-	-	-	-
C (2p3s ^1^P_1_→2p^2 1^D_2_) λ=193.1 nm	1.1 (0.3)	1.5	1.0 (0.2)	1.8	2.4 (0.5)	3.3	-	-	-	-	-	-
C (2p3s ^1^P_1_→2p^2 1^S_0_) λ=247.9 nm	0.4 (0.1)		0.8 (0.4)		0.9 (0.3)		-		-		-	
C_2_ Δν = 0, 1	-	-	-	-	13.3 (1.0)	13.3	-	-	-	-	-	-
He	-	-	-	-	1.6 (0.4)	1.6	-	-	-	-	-	-

^†^ Measured at 1000 eV [32].

**Table 3:** Simple thermochemical estimation of the lowest threshold energies (*E*_TH_) for reactions producing the most intense excited fragments in the collisions of H^+^/H_2_^+^ with pyridine. Reactants are assumed in their ground states. Note that CT—charge-transfer, DE—dissociative excitation, DI—dissociative ionization, and DP—direct dissociative excitation of a projectile.

No.	Reactants	Products	*E*_TH_ [eV]	Collisional Process
1.	H^+^ + C_5_H_5_N	H + C_5_H_5_N^+^	−4.40	CT
2.		H(*n* = 4) + C_5_H_5_N^+^	8.35	CT
3.		H + C_5_H_4_N^+^ + H(*n* = 4)	13.13	CT
4.		H + NCCHCHCHCH^+^ + H(*n* = 4)	14.24	CT
5.		H^+^ + C_5_H_4_N + H(*n* = 4)	17.53	DE
6.		H^+^ + NCCHCHCHCH + H(*n* = 4)	18.64	DE
7.		H^+^ + C_5_H_4_N^+^ + H(*n* = 4)	27.75	DI
8.		H + NCCHCH^+^ + CH(A^2^Δ)+ CH_2_	9.48	CT
9.		H + NCCHCHCH^+^ + CH(A^2^Δ) + H	9.61	CT
10.		H^+^ + NCCHCH + CH(A^2^Δ)+ CH_2_	13.88	DE
11.		H^+^ + NCCHCHCH + CH(A^2^Δ) + H	14.01	DE
12.		H^+^ + C_3_H_3_N^+^ + CH(A^2^Δ) + CH	26.68	DI
13.		H + CN(B^2^Σ^+^) + CHCHCHCH_2_^+^	5.17	CT
14.		H + CN(B^2^Σ^+^) + H + CH_2_CCHCH^+^	7.00	CT
15.		H^+^ + CN(B^2^Σ^+^) + CHCHCHCH_2_	9.54	DE
16.		H^+^ + CN(B^2^Σ^+^) + H + CH_2_CCHCH	11.40	DE
17.		H^+^ + CN(B^2^Σ^+^) + H+C_4_H_4_^+^	22.61	DI
18.	H_2_^+^ + C_5_H_5_N	H_2_ + C_5_H_5_N^+^	−6.23	CT
19.		H + H(*n* = 4) + C_5_H_5_N^+^	9.17	CT
20.		H_2_ + C_5_H_4_N^+^ + H(*n* = 4)	11.30	CT
21.		H_2_ + NCCHCHCHCH^+^ + H(*n* = 4)	12.41	CT
22.		H^+^ + H(*n*=4)+C_5_H_5_N	17.23	DP
23.		H_2_^+^+C_5_H_4_N+H(*n* = 4)	17.53	DE
24.		H_2_^+^ + NCCHCHCHCH+H(*n* = 4)	18.64	DE
25.		H_2_^+^ + C_5_H_4_N^+^ + H(*n* = 4)	27.75	DI
26.		H_2_ + NCCHCH^+^ + CH(A^2^Δ) + CH_2_	7.65	CT
27.		H_2_ + NCCHCHCH^+^ + CH(A^2^Δ) + H	7.78	CT
28.		H_2_^+^ + NCCHCH + CH(A^2^Δ) + CH_2_	13.88	DE
29.		H_2_^+^ + NCCHCHCH + CH(A^2^Δ) + H	14.01	DE
30.		H_2_^+^ + C_3_H_3_N^+^ + CH(A^2^Δ)+CH	26.68	DI
31.		H_2_ + CN(B^2^Σ^+^) + CHCHCHCH_2_^+^	3.31	CT
32.		H_2_ + CN(B^2^Σ^+^) + H + CH_2_CCHCH^+^	5.17	CT
33.		H_2_^+^ + CN(B^2^Σ^+^) + CHCHCHCH_2_	9.54	DE
34.		H_2_^+^ + CN(B^2^Σ^+^) + H + CH_2_CCHCH	11.40	DE
35.		H_2_^+^ + CN(B^2^Σ^+^) + H + C_4_H_4_^+^	22.61	DI

## Data Availability

The data presented in this study are available on request from the corresponding author.

## References

[1] Fulvio D., Potapov A., He J., Henning T. (2021). Astrochemical Pathways to Complex Organic and Prebiotic Molecules: Experimental Perspectives for In Situ Solid-State Studies. Life.

[2] Vasconcelos F.A., Pilling S., Agnihotri A., Rothard H., Boduch P. (2020). Methylenimine and cyanomethanimine synthesis from ion irradiation of N_2_-CH_4_ ice: Implication on the formation of prebiotic molecules in outer solar system bodies. Icarus.

[3] Kaiser R.I., Hansen N. (2021). An Aromatic Universe—A Physical Chemistry Perspective. J. Phys. Chem. A.

[4] Cooke I.R., Sims I.R. (2019). Experimental Studies of Gas Phase Reactivity in Relation to Complex Organic Molecules in Star-Forming Regions. ACS Earth Space Chem..

[5] Rothard H., Domaracka A., Boduch P., Palumbo M.E., Strazzulla G., Da Silveira E.F., Dartois E. (2017). Modification of ices by cosmic rays and solar wind. J. Phys. B Atom. Mol. Opt. Phys..

[6] Larsson M., Geppert W.D., Nyman G. (2012). Ion chemistry in space. Rep. Prog. Phys..

[7] Ali A., Sittler E.C.J., Chornay D., Rowe B.R., Puzzarini C. (2015). Organic chemistry in Titan’s upper atmosphere and its astrobiological consequences: I. Views towards Cassini plasma spectrometer (CAPS) and ion neutral mass spectrometer (INMS) experiments in space. Planet. Space Sci..

[8] Semo N.M., Koski W.S. (1984). Some ion–molecule reactions pertinent to combustion. J. Phys. Chem..

[9] Fialkov A.B. (1997). Investigations on ions in flames. Prog. Energy Combust. Sci..

[10] Skuratov V.A., Gun K.J., Stano J., Zagorski D.L. (2006). In situ luminescence as monitor of radiation damage under swift heavy ion irradiation. Nucl. Instrum. Methods Phys. Res. Sect. B.

[11] Townsend P.D. (2012). Variations on the use of ion beam luminescence. Nucl. Instrum. Methods Phys. Res. Sect. B.

[12] Townsend P.D., Crespillo M.L. (2016). An ideal system for analysis and interpretation of ion beam induced luminescence. Phys. Procedia.

[13] Utke I., Hoffmann P., Melngailis J. (2008). Gas-Assisted Focused Electron Beam and Ion Beam Processing and Fabrication. J. Vac. Sci. Technol. B.

[14] He S., Tian R., Wu W., Li W.-D., Wang D. (2021). Helium-Ion-Beam Nanofabrication: Extreme Processes and Applications. Int. J. Extrem. Manuf..

[15] Hayles M.F., De Winter D.A.M. (2021). An introduction to cryo-FIB-SEM cross-sectioning of frozen, hydrated Life Science samples. J. Microsc..

[16] Veligura V., Hlawacek G., van Gastel R., Zandvliet H.J.W., Poelsema B. (2014). A high resolution ionoluminescence study of defect creation and interaction. J. Phys. Condens. Matter.

[17] Hlawacek G., Veligura V., van Gastel R., Poelsema B. (2014). Helium Ion Microscopy. J. Vac. Sci. Technol. B.

[18] de Vries J., Hoekstra R., Morgenstern R., Schlatholter T. (2003). Charge Driven Fragmentation of Nucleobases. Phys. Rev. Lett..

[19] Schlathölter T., Hoekstra R., Morgenstern R. (2004). Charge Driven Fragmentation of Biologically Relevant Molecules. Int. J. Mass Spectrom..

[20] Deng Z., Bald I., Illenberger E., Huels M.A. (2005). Beyond the Bragg Peak: Hyperthermal Heavy Ion Damage to DNA Components. Phys. Rev. Lett..

[21] Schlathölter T., Alvarado F., Bari S., Hoekstra R. (2006). Ion-Induced Ionization and Fragmentation of DNA Building Blocks. Phys. Scr..

[22] Zettergren H., Domaracka A., Schlathölter T., Bolognesi P., Diaz-Tendero S., Łabuda M., Tosic S., Maclot S., Johnsson P., Steber A. (2021). Roadmap on dynamics of molecules and clusters in the gas phase. Eur. Phys. J. D.

[23] Bibang P.C.J.A., Agnihotri A.N., Boduch P., Domaracka A., Kanuchova Z., Rothard H. (2021). Radiolysis of pyridine in solid water. Eur. Phys. J. D.

[24] Wasowicz T.J. (2021). Photon luminescence studies of tetrahydrofuran following trihydrogen cations impact in the 20–1000 eV energy range. Rom. Rep. Phys..

[25] Wasowicz T.J., Pranszke B. (2021). Optical spectroscopic studies of tetrahydrofuran fragmentation induced by collisions with dihydrogen cations. Acta Phys. Pol. A.

[26] Wasowicz T.J., Pranszke B. (2016). Interactions of protons with furan molecules studied by collision-induced emission spectroscopy at the incident energy range of 50–1000 eV. Eur. Phys. J. D.

[27] Wasowicz T.J., Pranszke B. (2016). Observation of the hydrogen migration in the cation-induced fragmentation of the pyridine molecules. J. Phys. Chem. A.

[28] Wasowicz T.J., Pranszke B. (2015). Fragmentation of Tetrahydrofuran Molecules by H^+^, C^+^, and O^+^ Collisions at the Incident energy Range of 25−1000 eV. J. Phys. Chem. A.

[29] Wasowicz T.J., Pranszke B. (2015). Charge transfer and formation of complexes in the He^+^ collisions with the furan molecules. J. Phys. Conf. Ser..

[30] Wasowicz T.J. (2015). Hydrogen migration observed in fragmentation of the pyridine molecules in collisions with the H^+^, H_2_^+^, He^+^ and He^++^ cations. J. Phys. Conf. Ser..

[31] Wasowicz T.J., Łabuda M., Pranszke B. (2019). Charge Transfer, Complexes Formation and Furan Fragmentation Induced by Collisions with Low-Energy Helium Cations. Int. J. Mol. Sci..

[32] Wasowicz T.J. (2020). Collision-induced luminescence spectra of pyridine bombarded by 1000 eV He^+^ cations. Res. Phys..

[33] Amaldi U., Kraft G. (2005). Radiotherapy with Beams of Carbon Ions. Rep. Prog. Phys..

[34] Tinganelli W., Durante M. (2020). Carbon Ion Radiobiology. Cancers.

[35] Thariat J., Valable S., Laurent C., Haghdoost S., Peres E.A., Bernaudin M., Sichel F., Lesueur P., Cesaire M., Petit E. (2019). Hadrontherapy Interactions in Molecular and Cellular Biology. Int. J. Mol. Sci..

[36] Cucinotta F., Durante M. (2006). Cancer risk from exposure to galactic cosmic rays: Implications for space exploration by human beings. Lancet Oncol..

[37] Durante M., Cucinotta F.A. (2011). Physical basis of radiation protection in space travel. Rev. Mod. Phys..

[38] Durante M., Cucinotta F.A. (2008). Heavy Ion Carcinogenesis and Human Space Exploration. Nat. Rev. Cancer.

[39] Wasowicz T.J., Kivimäki A., Catone D., Richter R. (2020). Vacuum ultraviolet photoionization and ionic fragmentation of the isoxazole molecules. Int. J. Mass Spectrom..

[40] Kivimäki A., Stråhlman C., Wasowicz T.J., Kettunen J.A., Richter R. (2016). Yields and Time-of-Flight Spectra of Neutral High-Rydberg Fragments at the K Edges of the CO_2_ Molecule. J. Phys. Chem. A..

[41] Kivimäki A., Wasowicz T.J., Richter R. (2021). Soft X-ray Induced Production of Neutral Fragments in High-Rydberg States at the O 1s Ionization Threshold of the Water Molecule. J. Phys. Chem. A..

[42] McConkey J.W., Malone C.P., Johnson P.V., Winstead C., McKoy V., Kanik I. (2008). Electron Impact Dissociation of Oxygen-Containing Molecules. A Critical Review. Phys. Rep..

[43] Hatano H. (1999). Interaction of Vacuum Ultraviolet Photons with Molecules. Formation and Dissociation Dynamics of Molecular Superexcited States. Phys. Rep..

[44] Linert I., Lachowicz I., Wasowicz T.J., Zubek M. (2010). Fragmentation of Isoxazole Molecules by Electron Impact in the Energy Range 10−85 eV. Chem. Phys. Lett..

[45] Wasowicz T.J., Linert I., Lachowicz I., Zubek M. (2011). Electron impact fragmentation of pyrrole molecules studied by fluorescence emission spectroscopy. Photonics Lett. Pol..

[46] Hein J.D., Al-Khazraji H., Tiessen C.J., Lukic D., Trocchi J.A., McConkey J.W. (2013). Excited atomic fragments following electron dissociation of pyrimidine. J. Phys. B At. Mol. Opt. Phys..

[47] Erdevdi N.M., Zvenigorodskii V.V., Shpenik O.B., Romanova L.G. (2013). Excitation of Adenine Molecules by Slow Electrons. Opt. Spectrosc..

[48] Shpenik O.B., Erdevdy N.M., Zvenighorodsky V.V., Romanova L.G. (2013). Luminescence of cytosine vapor excited by slow electrons. J. Appl. Spectrosc..

[49] Hein J.D., Al-Khazraji H., Tiessen C.J., Lukic D., Trocchi J.A., McConkey J.W. (2016). VUV study of electron impact dissociative excitation of thymine. J. Phys. B At. Mol. Opt. Phys..

[50] Trocchi J.A., Dech J., Kedzierski W., McConkey J.W. (2019). Production of excited H–atoms in electron collisions with adenine. J. Phys. B At. Mol. Opt. Phys..

[51] Erdevdi N.M., Bulhakova A.I., Shpenik O.B., Zavilopulo A.N. (2020). Electron-Impact-Induced Excitation of Glutamine Molecules. Tech. Phys. Lett..

[52] Shpenik O.B., Maslyuk V.T., Zavilopulo A.N., Erdevdi N.M., Bulhakova A.I., Megela I.G. (2021). Electron impact excitation of glutamine molecules irradiated with an M-30 microtron with an energy of 11.5 MeV. J. Phys. B At. Mol. Opt. Phys..

[53] Wasowicz T.J., Kivimaki A., Dampc M., Coreno M., De Simone M., Zubek M. (2011). Photofragmentation of Tetrahydrofuran Molecules in the Vacuum-Ultraviolet Region via Superexcited States Studied by Fluorescence Spectroscopy. Phys. Rev. A.

[54] Wasowicz T.J., Kivimaki A., Coreno M., Zubek M. (2012). Superexcited States in the Vacuum-Ultraviolet Photofragmentation of Isoxazole Molecules. J. Phys. B At. Mol. Opt. Phys..

[55] Zubek M., Wasowicz T.J., Dąbkowska I., Kivimaki A., Coreno M. (2014). Hydrogen Migration in Formation of NH(A^3^Π) Radicals Via Superexcited States in Photodissociation of Isoxazole Molecules. J. Chem. Phys..

[56] Wasowicz T.J., Kivimaki A., Coreno M., Zubek M. (2014). Formation of CN(B^2^Σ^+^) Radicals in the Vacuum-Ultraviolet Photodissociation of Pyridine and Pyrimidine Molecules. J. Phys. B At. Mol. Opt. Phys..

[57] Wasowicz T.J., Kivimäki A., Coreno M., Zubek M. (2015). Hydrogen migration in photodissociation of the pyridine molecules. J. Phys. Conf. Ser..

[58] Wasowicz T.J., Dabkowska I., Kivimäki A., Coreno M., Zubek M. (2017). Elimination and migration of hydrogen in the vacuum-ultraviolet photodissociation of pyridine molecules. J. Phys. B Atom. Mol. Opt. Phys..

[59] Ozga C., Reiß P., Kielich W., Klumpp S., Knie A., Ehresmann A. (2015). Fluorescence cascades after excitation of Xe II 5p^4^6p satellite states by synchrotron radiation. J. Phys. B Atom. Mol. Opt. Phys..

[60] Wasowicz T.J., Kivimäki A., Stupar M., Coreno M. (2018). Study of ultraviolet-visible fluorescence emission following resonant Auger decay of the 2p^-1^nl core-excited states of argon atoms. J. Elec. Spectrosc. Rel. Phenom..

[61] Reiß P., Schmidt P., Ozga C., Kniel A., Ehresmann A. (2015). Dispersed fluorescence spectrometry from the VIS to VUV spectral range for experiments at heavy-ion storage facilities. Phys. Scr..

[62] Kivimäki A., Alvarez-Ruiz J., Wasowicz T.J., Callegari C., de Simone M., Alagia M., Richter R., Coreno M. (2011). O 1s excitation and ionization processes in the CO_2_ molecule studied via detection of low-energy fluorescence emission. J. Phys. B Atom. Mol. Opt. Phys..

[63] Kojima T., Aihara H., Kodashima Y., Makishima H., Nakiri S., Takada S., Shimada H., Ukai M., Ozga C., Holzapfel X. (2019). Novel analytical study for reaction intermediates in the primary radiation interaction of DNA using a synchrotron radiation-induced luminescence spectroscopy. Radiat. Protec. Dosim..

[64] Hans A., Schmidt P., Ozga C., Hartmann G., Holzapfel X., Ehresmann A., Knie A. (2018). Extreme Ultraviolet to Visible Dispersed Single Photon Detection for Highly Sensitive Sensing of Fundamental Processes in Diverse Samples. Materials.

[65] Rashid S., Sit A., West B., Mayer P.M. (2017). Colliding the hydrocarbon building blocks of astrochemical polycyclic aromatic hydrocarbons with 8 keV and ions: Luminescence from methane, acetylene, benzene and naphthalene. Chem. Phys. Lett..

[66] Baumann M., Baxendale I. (2013). An overview of the synthetic routes to the best selling drugs containing 6-membered heterocycles. Beilstein J. Org. Chem..

[67] Kiuru P., Yli-Kauhaluoma J., Majumdar K., Chattopadhyay S.K. (2011). Pyridine and Its Derivatives. Heterocycles in Natural Product Synthesis.

[68] Callaghan M.P., Smith K.E., Cleaves H.J., Ruzicka J., Stern J.C., Glavin D.P., House C.H., Dworkin J.P. (2011). Carbonaceous meteorites contain a wide range of extraterrestrial nucleobases. Proc. Natl. Acad. Sci. USA.

[69] Smith K.E., Callahan M.P., Gerakines P.A., Dworkin J.P., House C.H. (2014). Investigation of pyridine carboxylic acids in CM2 carbonaceous chondrites: Potential precursor molecules for ancient coezymes. Geochim. Cosmichim. Acta.

[70] Charnley S.B., Kuan Y.-J., Huang H.-C., Botta O., Butner H.M., Cox N., Despois D., Ehrenfreund P., Kisiel Z., Lee Y.-Y. (2005). Astronomical Searches for Nitrogen Heterocycles. Adv. Space Res..

[71] Zdanovskaia M.A., Dorman P.M., Orr V.L., Owen A.N., Kougias S.M., Esselman B.J., Woods R.C., McMahon R.J. (2021). Rotational Spectra of Three Cyanobutadiene Isomers (C_5_H_5_N) of Relevance to Astrochemistry and Other Harsh Reaction Environments. J. Am. Chem. Soc..

[72] Zhai Y.-E., Shi D.-Q. (2013). Synthesis and Herbicidal Activity of 2-Alkyl(aryl)-4-amino-3-[alkyl(alkoxy)carbonyl]- 5-cyano-6-[(3-trifluoromethyl)phenoxy]-pyridines. J. Heterocycl. Chem..

[73] Fondren L.D., McLain J., Jackson D.M., Adams N.G., Babcock L.M. (2007). Studies of Reactions of a Series of Ions With Nitrogen Containing Heterocyclic Molecules Using a Selected Ion Flow Tube. Int. J. Mass Spectrom..

[74] Lucas M., Thomas A.M., Kaiser R.I., Bashkirov E.K., Azyazov V.N., Mebel A.M. (2018). Combined Experimental and Computational Investigation of the Elementary Reaction of Ground State Atomic Carbon (C; ^3^P_j_) with Pyridine (C_5_H_5_N; X^1^A_1_) via Ring Expansion and Ring Degradation Pathways. J. Phys. Chem. A.

[75] Recio P., Marchione D., Caracciolo A., Murray V.J., Mancini L., Rosi M., Casavecchia P., Balucani N. (2021). A crossed molecular beam investigation of the N(2D) + pyridine reaction and implications for prebiotic chemistry. Chem. Phys. Lett..

[76] Feldman U., Landi E., Schwadron N.A. (2005). On the sources of fast and slow solar wind. J. Geophys. Res..

[77] Zeitlin C., Hassler D.M., Cucinotta F.A., Ehresmann B., Wimmer-Schweingruber R.F., Brinza D.E., Kang S., Weigle G., Böttcher S., Böhm E. (2013). Measurements of Energetic Particle Radiation in Transit to Mars on the Mars Science Laboratory. Science.

[78] Yogo A., Sato K., Nishikino M., Mori M., Teshima T., Numasaki H., Murakami M., Demizu Y., Akagi S., Nagayama S. (2009). Application of laser-accelerated protons to the demonstration of DNA double-strand breaks in human cancer cells. Appl. Phys. Lett..

[79] Allison R.R., Sibata C., Patel R. (2013). Future radiation therapy: Photons, protons and particles. Future Oncol..

[80] Mein S., Tessonnier T., Kopp B., Harrabi S., Abdollahi A., Debus J., Haberer T., Mairani A. (2021). Spot-Scanning Hadron Arc (SHArc) Therapy: A Study With Light and Heavy Ions. Adv. Rad. Oncol..

[81] Loeffler J.S., Durante M. (2013). Charged particle therapy—optimization, challenges and future directions. Nat. Rev. Clin. Oncol..

[82] Schulz-Ertner D., Jäkel O., Schlegel W. (2006). Radiation Therapy with Charged Particles. Semin. Radiat. Oncol..

[83] Tsujii H., Kamada T., Baba M., Tsuji H., Kato H., Kato S., Yamada S., Yasuda S., Yanagi T., Kato H. (2008). Clinical advantages of carbon-ion radiotherapy. New J. Phys..

[84] Luque J., Crosley D.R. (1999). Lifbase: Database and Spectral Simulation (Version 1.5), SRI International Report MP 99-009. https://www.sri.com/case-studies/lifbase-spectroscopy-tool/.

[85] Brzozowski J., Bunker P., Elander N., Erman P. (1976). Predissociation efects in the A, B, and C states of CH and the interstellar formation rate of CH via inverse predissociation. Astrophys. J..

[86] Zachwieja M. (1995). New Investigations of the A^2^Δ-X^2^Π Band System in the CH Radical and a New Reduction of the Vibration-Rotation Spectrum of CH from the ATMOS Spectra. J. Mol. Spectroc..

[87] Luque J., Crosley D.R. (1996). Electronic transition moment and rotational transition probabilities in CH. I. A^2^Δ-X^2^Π system. J. Chem. Phys..

[88] Cerny D., Bacis R., Guelavchvili G., Roux F. (1978). Extensive analysis of the red system of the CN molecule with a high resolution Fourier Spectrometer. J. Mol. Spectrosc..

[89] Ito H., Ozaki Y., Suzuki K., Kondow T., Kuchitsu K. (1988). Analysis of the *B*^2^Σ^+^ ~ *A*^2^Π*_i_* perturbations in the CN(*B*^2^Σ^+^-*X*^2^Σ^+^) main band system: I. Molecular constants for B2Σ+ and A2Πi. J. Mol. Spectrosc..

[90] Mogyorosi K., Sarosi K., Chikan V. (2020). Direct Production of CH(A^2^Δ) Radical from Intense Femtosecond Near-IR Laser Pulses. J. Phys. Chem. A.

[91] Mogyorosi K., Sarosi K., Seres I., Jojart P., Fule M., Chikan V. (2020). Formation of CN Radical from Nitrogen and Carbon Condensation and from Photodissociation in Femtosecond Laser-Induced Plasmas: Time-Resolved FT-UV−Vis Spectroscopic Study of the Violet Emission of CN Radical. J. Phys. Chem. A.

[92] Trentelman K.A., Kable S.H., Moss D.B., Houston P.L. (1989). Photodissociation dynamics of acetone at 193 nm: Photofragment internal and translational energy distributions. J. Chem. Phys..

[93] Lin M.-F., Dyakov Y.A., Tseng C.-M., Mebel A.M., Lin S.H., Lee Y.T., Ni C.-K. (2005). Photodissociation dynamics of pyridine. J. Chem. Phys..

[94] Chikan V., Fournier F., Leone S.R., Nizamov B. (2006). State-Resolved Dynamics of the CH(A^2^Δ) Channels from Single and Multiple Photon Dissociation of Bromoform in the 10–20 eV Energy Range. J. Phys. Chem. A.

[95] Pei L., Farrar J.M. (2012). Imaging ion-molecule reactions: Charge transfer and C–N bond formation in the C^+^ + NH3 system. J. Chem. Phys..

[96] Bethe H.E., Salpeter E.E. (1977). Quantum Mechanics of One-and Two-Electron Atoms.

[97] Windholz L., Winklhofer E., Drozdowski R., Kwela J., Wasowicz T.J., Heldt J. (2008). Stark effect of atomic Helium second triplet series in electric fields up to 1600 kV/cm. Phys. Scr..

[98] Windholz L., Drozdowski R., Wasowicz T.J., Kwela J. (2005). Anticrossing effects in Stark spectra of helium. Proc. SPIE.

[99] Windholz L., Drozdowski R., Wasowicz T., Kwela J. (2006). Stark effect in He I in extremely high electric field. Opt. Appl..

[100] Windholz L., Wasowicz T.J., Drozdowski R., Kwela J. (2012). Stark effect of atomic Helium singlet lines. J. Opt. Soc. Am. B.

[101] Hoekstra R., de Heer F.J., Morgenstern R. (1991). State-selective electron capture in collisions of He^2+^ with H. J. Phys. B Mol. Opt. Phys..

[102] Ramsey N.F., Dunning F.B., Hulet R.D. (1996). Thermal Beam Sources. Atomic, Molecular, and Optical Physics: Atoms and Molecules.

[103] Bacchus-Montabonel M.-C. (2013). Looking at Radiation Damage on Prebiotic Building Blocks. J. Phys. Chem. A.

[104] Akbulut M., Sack N.J., Madey T.E. (1997). Elastic and Inelastic Processes in the Interaction of l-10 eV Ions with Solids: Ion Transport through Surface Layers. Surf. Sci. Rep..

[105] Alvarado F., Bari S., Hoekstra R., Schlathölter T. (2006). Quantification of Ion-Induced Molecular Fragmentation of Isolated 2-Deoxy-D-ribose Molecules. Phys. Chem. Chem. Phys..

[106] Bowen R.D. (1991). Ion-Neutral Complexes. Acc. Chem. Res..

[107] Sladek V., Skorna P., Poliak P., Lukes V. (2015). The ab initio Study of Halogen and Hydrogen σN-Bonded Para-Substituted Pyridine (X2/XY/HX) Complexes. Chem. Phys. Lett..

[108] Wren S.W., Vogelhuber K.M., Garver J.M., Kato S., Sheps L., Bierbaum V.M., Lineberger W.C. (2012). C–H Bond Strengths and Acidities in Aromatic Systems: Effects of Nitrogen Incorporation in Mono-, Di-, and Triazines. J. Am. Chem. Soc..

[109] Liu J., Salumbides E.J., Hollenstein U., Koelemeij J.C.J., Eikema K.S.E., Ubachs W., Merkt F. (2009). Determination of the Ionization and Dissociation Energies of the Hydrogen Molecule. J. Chem. Phys..

[110] Jiao C.Q., DeJoseph J.C.A., Lee R., Garscadden A. (2006). Kinetics of electron impact ionization and ion-molecule reactions of pyridine. Int. J. Mass Spectrom..

[111] Linstrom P.J., Mallard W.G., NIST Chemistry WebBook (2021). NIST Standard Reference Database Number 69.

[112] Smialek M.A., MacDonald M.A., Ptasinska S., Zuin L., Mason N.J. (2016). Photoelectron and Threshold Photoelectron Valence Spectra of Pyridine. Eur. Phys. J. D.

[113] Blanksby S.J., Ellison G.B. (2003). Bond Dissociation Energies of Organic Molecules. Acc. Chem. Res..

[114] Vall-llosera G., Coreno M., Erman P., Huels M.A., Jakubowska K., Kivimäki A., Rachlew E., Stankiewicz M. (2008). VUV photoionization of free azabenzenes: Pyridine, pyrazine, pyrimidine, pyridazine, and s-triazine. Int. J. Mass Spectrom..

[115] Gappa A., Herpers E., Herrmann R., Huelsewede V., Kappert W., Klar M., Kirmse W. (1995). Ion−Molecule Complexes in 1,2-Alkyl Shifts. J. Am. Chem. Soc..

[116] Ijaz W., Gregg Z., Barnes G.L. (2013). Complex Formation during SID and Its Effect on Proton Mobility. J. Phys. Chem. Lett..

[117] Amunugama R., Rodgers M.T. (2000). Absolute Alkali Metal Ion Binding Affinities of Several Azines Determined by Threshold Collision-Induced Dissociation and ab initio Theory. Int. J. Mass Spectrom..

[118] Wang Z., Zheng B., Yu X., Li X., Yi P. (2010). Structure, Properties, and Nature of the Pyridine-XY (X, Y = F, Cl, Br) Complexes: An ab initio Study. J. Chem. Phys..

[119] Wu J., Yan H., Jin Y., Dai G., Zhong A. (2010). Characteristics and Nature of the Intermolecular Interactions between Pyridine and Various Hydrides: A Theoretical Study. J. Mol. Struct. THEOCHEM.

[120] El-Shall M.S., Ibrahim Y., Alsharaeh E.H., Meot-Ner M., Watson S.P. (2009). Reactions between Aromatic Hydrocarbons and Heterocycles: Covalent and Proton-bond Dimer Cations of Benzene/Pyridine. J. Am. Chem. Soc..

[121] Feng J.-Y., Lee Y.-P., Witek H.A., Ebata T. (2021). Vacuum Ultraviolet Photoionization Induced Proton Migration and Formation of a New C−N Bond in Pyridine Clusters Revealed by Infrared Spectroscopy and Mass Spectrometry. J. Phys. Chem. Lett..

[122] Rap D.B., Marimuthu A.N., Redlich B., Brünken S. (2020). Stable Isomeric Structures of the Pyridine Cation (C_5_H_5_N^•^^+^) and Protonated Pyridine (C_5_H_5_NH^•+^) elucidated by Cold Ion Infrared Spectroscopy. J. Mol. Spectrosc..

[123] Mackie J.C., Colket M.B., Nelson P.F. (1990). Shock Tube Pyrolysis of Pyridine. J. Phys. Chem..

[124] Kiefer J.H., Zhang Q., Kern R.D., Yao J., Jursic B. (1997). Pyrolyses of Aromatic Azines: Pyrazine, Pyrimidine, and Pyridine. J. Phys. Chem. A.

[125] Hore N.R., Russell D.K. (1998). Radical Pathways in the Thermal Decomposition of Pyridine and Diazines: A Laser Pyrolysis and Semi-empirical Study. J. Chem. Soc. Perkin Trans. 2.

[126] Ni C.-K., Tseng C.-M., Lin M.F., Dyakov Y.A. (2007). Photodissociation Dynamics of Small Aromatic Molecules Studied by Multimass Ion Imaging. J. Phys. Chem. B.

[127] Zhong D., Diau E.W.-G., Bernhardt T., De Feyter S., Roberts J.D., Zewail A.H. (1998). Femtosecond dynamics of valence-bond isomers of azines: Transition states and conical intersections. Chem. Phys. Lett..

[128] Lobastov V.A., Srinivasan R., Goodson B.M., Ruan C.-Y., Feenstra J.S., Zewail A.H. (2001). Ultrafast Diffraction of Transient Molecular Structures in Radiationless Transitions. J. Phys. Chem. A.

[129] Ehbrecht A., Kowalski A., Ottinger C. (1998). Hot-atom chemiluminescence: A beam study of the reactions C(^3^P) + H_2_→CH (A_2_Δ, B^2^Σ^−^, C_2_Σ^+^) + H. Chem. Phys. Lett..

[130] Drozdowski R., Kowalski A. (2018). Luminescence cross sections in the low-energy collisions of H^+^, H_2_^+^, and H_3_^+^ ions with H_2_. Eur. Phys. J. D.

[131] Glenewinkel-Meyer T., Muller B., Ottinger C., Tischer H. (1988). Measurement of the HCl^+^(*A* ^2^Σ^+^–*X* ^2^Π) electronic transition moment using quasiresonant charge transfer at low energy. J. Chem. Phys..

[132] Pyridine. https://www.sigmaaldrich.com/PL/pl/product/sial/270970.

